# Liability of Health Professionals Using Sensors, Telemedicine and Artificial Intelligence for Remote Healthcare

**DOI:** 10.3390/s24113491

**Published:** 2024-05-28

**Authors:** Marie Geny, Emmanuel Andres, Samy Talha, Bernard Geny

**Affiliations:** 1 Joint Research Unit-UMR 7354, Law, Religion, Business and Society, University of Strasbourg, 67000 Strasbourg, France; mariegeny.mg@gmail.com; 2 Biomedicine Research Center of Strasbourg (CRBS), UR 3072, “Mitochondria, Oxidative Stress and Muscle Plasticity”, University of Strasbourg, 67000 Strasbourg, France; emmanuel.andres@chru-strasbourg.fr (E.A.); samy.talha@chru-strasbourg.fr (S.T.); 3 Faculty of Medicine, University of Strasbourg, 67000 Strasbourg, France; 4 Department of Internal Medicine, University Hospital of Strasbourg, 67091 Strasbourg, France; 5 Department of Physiology and Functional Explorations, University Hospital of Strasbourg, 67091 Strasbourg, France

**Keywords:** sensors, telemedicine, artificial intelligence, healthcare, professional liability, responsibility, civil and tort law, ethics

## Abstract

In the last few decades, there has been an ongoing transformation of our healthcare system with larger use of sensors for remote care and artificial intelligence (AI) tools. In particular, sensors improved by new algorithms with learning capabilities have proven their value for better patient care. Sensors and AI systems are no longer only non-autonomous devices such as the ones used in radiology or surgical robots; there are novel tools with a certain degree of autonomy aiming to largely modulate the medical decision. Thus, there will be situations in which the doctor is the one making the decision and has the final say and other cases in which the doctor might only apply the decision presented by the autonomous device. As those are two hugely different situations, they should not be treated the same way, and different liability rules should apply. Despite a real interest in the promise of sensors and AI in medicine, doctors and patients are reluctant to use it. One important reason is a lack clear definition of liability. Nobody wants to be at fault, or even prosecuted, because they followed the advice from an AI system, notably when it has not been perfectly adapted to a specific patient. Fears are present even with simple sensors and AI use, such as during telemedicine visits based on very useful, clinically pertinent sensors; with the risk of missing an important parameter; and, of course, when AI appears “intelligent”, potentially replacing the doctors’ judgment. This paper aims to provide an overview of the liability of the health professional in the context of the use of sensors and AI tools in remote healthcare, analyzing four regimes: the contract-based approach, the approach based on breach of duty to inform, the fault-based approach, and the approach related to the good itself. We will also discuss future challenges and opportunities in the promising domain of sensors and AI use in medicine.

## 1. Introduction

The breakthrough of sensor development and artificial intelligence (AI) has already transformed our healthcare system. Sensors with high levels of accuracy in detecting clinically pertinent parameters are now widely used, allowing for remote healthcare at a time of severe shortage of medical and paramedical caregivers. Use of sensors in telemedicine already permits the remote monitoring of many key physiological parameters, including systemic blood pressure, heart rate, oxygen saturation, and glycaemia. Recent technologies, such as AI, edge computing, and Internet of Things (IoT), will likely be the cornerstone of new healthcare systems’ evolution [1]. Sensor devices can be associated with AI, which is defined by the Organization for Economic Co-operation and Development (OECD) as a “ machine-based system that, for explicit or implicit objectives, infers, from the input it receives, how to generate outputs such as predictions, content, recommendations, or decision that can influence physical or virtual environments” [2]. Sensors and AI systems can operate with a wide range of degrees of autonomy, which distinguishes them from other technologies in healthcare. Use of sensors and AI is exponentially increasing in almost all aspects of medical care, ranging from biology to clinical approaches, including heart failure, cancer, radiology, electrocardiogram analysis, and even the investigation of patients’ movement, which is of critical importance, for instance, in preventing falls [3,4,5,6,7,8,9].

Besides granting each patient better diagnosis and therapeutic proposals, the public health implications of AI and sensors are significant. With sensors and AI in healthcare comes the promise of enhancing the efficiency and effectiveness of public health interventions, leading to better health outcomes on a population level [10]. Accordingly, AI and sensors can help to improve patients’ care pathways. Such an approach positively affects public health outcomes. Thus, the use of remote monitoring for vital parameters and symptoms allowed for early hospital discharge of patients hospitalized for COVID-19, even those requiring oxygen therapy. The reduction in hospital stay likely resulted in more beds being available for other patients, which is critical during sanitary crises [11].

While acknowledging the potential beneficial effects of sensors and AI use on public health, questions arise about their generalization, data availability, and cost-saving potential. 

The generalizability of AI findings across different populations is a core issue. To ensure that AI findings are generalizable, principles regarding the treatment of data should be followed. First, data have to be lawful, fair, transparent, and obtained with security and safety [12]. In addition, the diversity and representativeness of the data used to train AI models are key determinants of generalizability. 

Data availability is another key component of the reproducibility of AI research studies because AI models may have variable performance based on the datasets used. However, less than one-third of the articles share code (code sharing) and adequately document methods [13]. With this knowledge, it is difficult to establish that AI findings are always generalizable across different populations. Indeed, data from one population may not accurately capture the nuances of other populations. 

Financial impact analysis of AI and sensor technology is essential for patients and hospital management. AI systems are already used in hospitals for administrative tasks, such as billing and coding, leading to operational efficiencies and reduced overhead costs. The Montreal hospital developed a monitoring solution to filter the mass of scientific information related to COVID. The aim was to reduce the time and money spent sifting through the 2000 or so scientific articles published each week and to establish their quality [14]. Further, in the study mentioning a chain of liability in a specific model with a nurse, it was stated that the data from the model could also serve as fuel to “build customized early warning systems and simultaneously to create algorithms for optimizing device performance” [15]. This type of analysis of the AI itself could lead to cost savings. Another example is the utilization of digital technologies for cataract screening in order to address the insufficiency of resources for the growing aging population. The authors used decision-analytic models to evaluate the cost-effectiveness of different strategies. They observed that digital technology-driven hierarchical screening (DH) is cost-effective as compared to no screening, tele screening, and AI screening in urban and rural China. This result provided an economic rationale for policymakers promoting public eye health in low- and middle-income countries [16].

In brief, sensors and AI have now been developed for almost all parts of healthcare, ranging from general organization and patient care pathways to specific investigations focused on cardiovascular, respiratory, ocular, or movement characteristics, etc. They therefore embrace many technological approaches [1].

However, although sensors and AI offer additional information that cannot always be obtained with classical parameters, pitfalls remain with doubts as to the reliability and analysis (black box) of the data, together with medico-legal and ethical issues. Thus, for instance, patient privacy, consent, data ownership, and accountability, support investigations on ethical and legal frameworks, guidelines, and policies necessary to govern the use of sensors and AI in healthcare. Transparency and fairness are needed to obtain the adherence of both patients and caregivers [1]. 

There have been attempts to categorize the various forms of AI. According to its capacities, AI has been classified into three main types [17]: artificial narrow intelligence (ANI) that can complete a specific task, artificial general intelligence (AGI) that can copy human intelligence by completing simultaneous tasks, and artificial super intelligence (ASI) that has the potential to outperform human intelligence.

**Figure 1:**
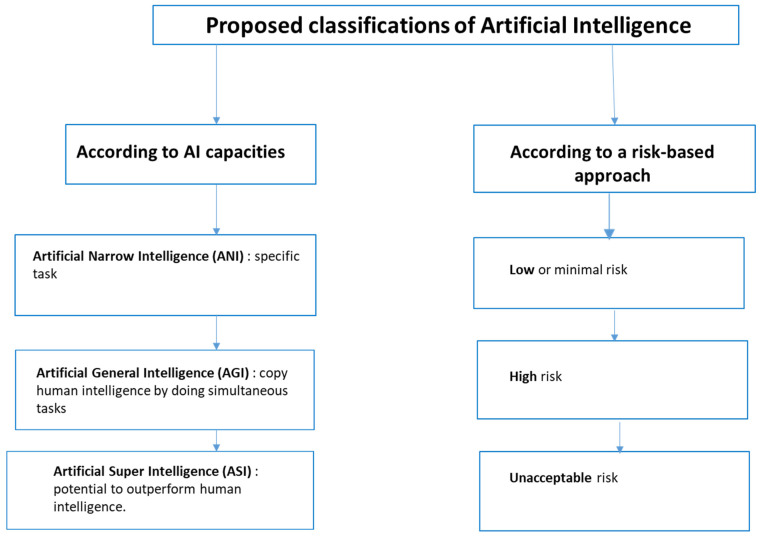
Tentative categorization of forms of AI.

In addition, in Europe, some authors retain a bipartite division utilizing weak AI, which refers to a program that focuses on tasks by following given instructions, and, by contrast, strong AI [2,18], which can learn and solve complex problems. Lastly, the European Union (EU) has categorized AI systems in the AI Act [10], employing a risk-based approach, resulting in a tripartite classification scaling from AI posing an unacceptable risk, a high risk, and a low or minimal risk (Figure 1). Here, the risk is evaluated in terms of potential impacts on the fundamental rights and safety of human beings [10]. In the context of healthcare, it encompasses the risks of patient harm but also the risks of a medical professional making the wrong decision by following a biased AI system, resulting in patient harm. Amongst these classifications, the one based on AI’s capacities might appear to be the more reliable, as the risk-based approach could be irrelevant if there is a change in society’s core values regarding to what degree the domain would be deemed risky. Nevertheless, the level of risk is already successfully used to grade the ethical rules to be fulfilled in the setting of clinical research. It is also used when analyzing the benefit–risk of sensors’ use.

It is critical to consider sensors’ and AI systems’ impact on healthcare in order to better understand the emerging challenges, especially since sensors associated with AI systems become progressively more autonomous in such a way that they can “operate independently and make decisions without human intervention” [19]. Regardless of the degree of autonomy of these systems, their output can be wrong, which could lead to patient harm and medical malpractice claims. Yet, the “legal standards for medical malpractice liability differ from country to country but share common principles” [20]. In France, medical liability has been defined as “the obligation for healthcare professional to compensate for the harm suffered by a patient” [21]. In the context of patient care, many healthcare professionals engage in “the patient-physician relationship”, in which they have legal obligation. The medical liability of health professional is based on specific legal grounds related to various situations (contractual relationship established with the patient, failure to provide information, or fault). Thus, attention needs to be paid to the ethical, regulatory, and social aspects of sensors and AI, which largely impact both professionals’ and citizens’ acceptance [22,23].

The objective of this review is to provide an analysis of the legal liability regimes existing for healthcare professionals using remote sensors, telemedicine, and/or artificial intelligence.

Besides considering technical and ethical acceptability and public health impacts, we aimed to address the need for more clarity and uniformity around the legal responsibilities of healthcare professionals using AI and sensors in practice and did so by comparing different jurisdictions’ legislation on medical liability in the context of emerging technology. 

In this respect, we present the French legal system and other examples from different countries to synthesize and compare legal approaches and present some of the best practices in terms of medical liability and AI devices.

## 2. Methods

We utilized a two-stage review of the literature.

(1) A stepwise systematic review process according to the Preferred Reporting Items for Systematic Reviews and Meta-Analyses (PRISMA) guidelines and focused on PubMed [24].

(2) An analysis of legal-general databases such as the World Legal Information Institute and a French open database named Hyper Articles Online (HAL) to find other eligible studies.

### 2.1. Selection Criteria

We followed inclusion and exclusion criteria during the literature search to identify relevant studies. All references investigating the relationship between “medical liability” OR “liability” on one hand AND “sensors” OR “telemedicine” OR “artificial intelligence” on the other hand were included in the analysis. We focused the research published between 1 January 2022 and 1 May 2024 and included all full-text publications.

### 2.2. Research Strategy and Data Collection Process

(1) Combining the relevant keywords previously presented, each with all others, we created a flow chart focused on PubMed, reporting recorded and excluded articles throughout the review process.

After the removal of articles with free full-text versions not available, duplicates, and retracted records, we screened the titles and abstracts of each reference and retrieved pertinent full-text articles. Additional publications found in the references list of the papers analyzed were also included.

(2) We also added specific data allowing for a better definition of the concepts and context of liability and sensors or artificial intelligence, investigating technical, ethical, and public health aspects.

## 3. Results

**Figure 2:**
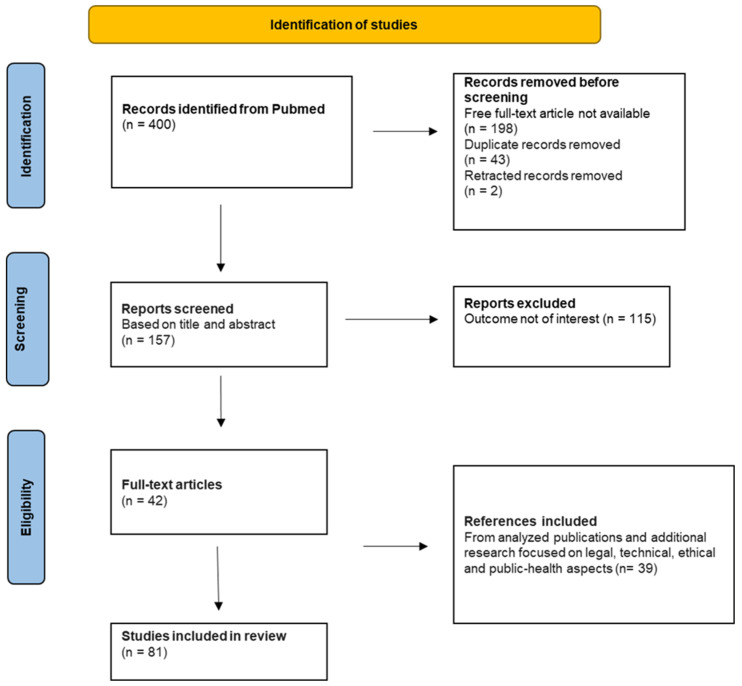
Flow-chart of the review.

The flow chart showing the identification, screening, and eligibility of the records is presented below (Figure 2). Data extraction led to the evaluation of 157 publications available in PubMed, of which 42 were included. Complementary research resulted in the inclusion of 39 papers. Ultimately, 81 publications were cited in the review.

## 4. Discussion

### 4.1. Unknown Liability Likely Limits Sensors and AI Use in Healthcare

#### 4.1.1. Healthcare Professional Perspective

While it is true that sensors and AI have the exponential potential to revolutionize the role of the doctor, this does not imply an imminent extinction of the profession in the years to come. Beyond their expertise, the doctor proves indispensable through their human qualities and remains essential in medical care that encompasses various forms of questions from the patient.

However, in the United States, the US Food and Drug Administration (FDA) has approved multiple AI-based tools, most of which include sensors tools. In consequence, health institutions have been deploying them in hospitals and other places. These systems support clinical decisions, such as recommending drugs or dosages or interpreting radiological images. Nevertheless, some systems communicate results or recommendations to the care team without being able to explain the underlying reasons for those results [20,25].

**Figure 3:**
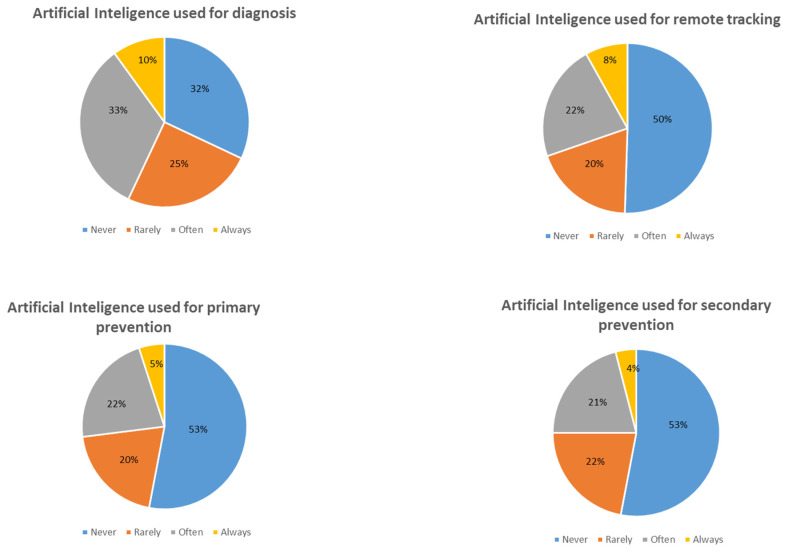
Percent of doctors using AI as part of their professional practice and the frequency of a specific use of a connected object for either diagnosis, remote tracking, primary, or secondary prevention.

In this regard, an absence of clarity around accountability for these solutions renders the healthcare professional reluctant toward the adoption and use of remote sensors and AI systems [26]. Indeed, in France, a study from MACSF (an insurer for healthcare professionals) and WITHINGS (creators of connected health devices) concluded that the professional use of health devices will remain “anecdotal until questions of responsibility of doctors, data security, and future of the patient-physician relationship” are clarified [27]. The study shows that amongst 1037 doctor members of MACSF, 43%, 30%, 27%, and 25% working with connected devices used them often or always to establish, respectively, either diagnosis, remote tracing, primary, or secondary prevention. According to the World Health Organization, primary prevention is the reduction of the incidence of a disease in a population (for example, a vaccination campaign likely to prevent infectious diseases). Secondary prevention aims to decrease the prevalence of a disease, which means to detect the disease at an early stage [28] (Figure 3 modified with permission from the MACSF and WITHINGS study). 

**Figure 4:**
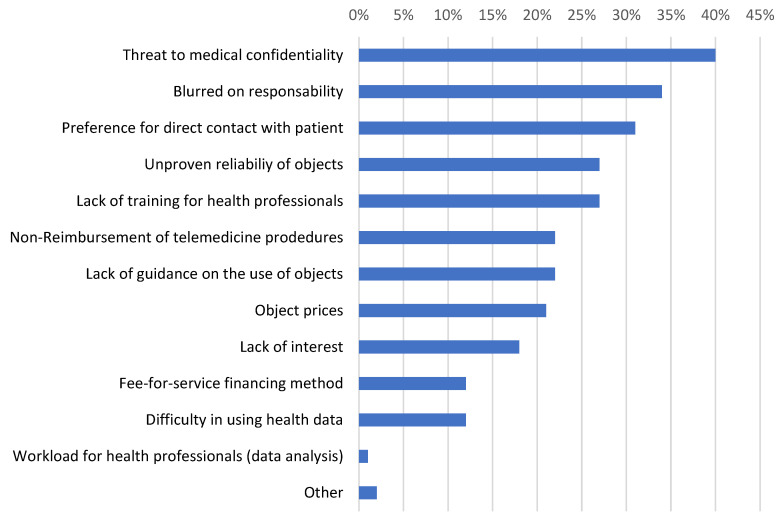
Current obstacles to the use of connected sensors in healthcare, according to doctors.

Further and importantly, more than one-third of doctors remained cautious about the applicable liability regime in the case of a tool they might have recommended ending up being implicated in the deterioration of a patient’s health (Figure 4). This is not specific to AI but might clearly generate a dilemma as to whether or not to use a surgical or medical tool with the balance of a potential benefit for many patients against a risk for some and medico-legal issues [29].

A recent study stated that 66% of caregivers (64% of doctors) believe that AI could allow them to allocate additional time to the patient. Nevertheless, 66% (60% of doctors) also express concerns about a deterioration of the bond of trust with their patients [30].

In the field of telemedicine using connected sensors, which covers teleconsultation, tele-expertise, remote medical monitoring, and remote assistance [31], the question of the distribution of responsibilities is one that needs to be asked. Indeed, in the case of tele-expertise, information must circulate between the requesting and the requested physician. The requesting physician keeps his or her independence of practice, and the tele-expert must consider the inherent limits of the practice. In case of shared error, there can be joint liability but also several liabilities for each. In the case of teleconsultation or tele-monitoring, the physician must remain vigilant about the information provided. With the development of telemedicine, liability may be envisaged for failure to use the procedure if available to the physician but also for using it in a faulty manner.

Another example exists in the field of radiology, where non-autonomous AI tools assist radiologists by showing the probability that an X-ray shows an abnormality. On the other hand, autonomous AI systems (or strong AI tending to progressively become autonomous) might “independently identify normal X-rays and generate reports, bypassing radiologists” [19]. Thus, when AI is autonomous, tension will arise about the responsibility, especially when the physicians struggle to understand how the system works or when they are forced to seek multiple ways of validating their decision to follow or reject AI recommendations [32]. 

According to Mezrich et al. [33], the professional use of sensors and AI creates new challenges, and the tort law applicable to these systems is not yet well developed. In the following discussion, we will explore the different regimens of medical liability in the face of AI in France and draw some parallels with other countries. Although potentially different depending on the country considered, these data might help one to better handle such an important parameter which can encourage or block professionals’ use of AI in healthcare, whatever its personal place in the overall care system of a country. However, before such an analysis, the patients’ view is important to consider.

#### 4.1.2. Patients’ Perspective

How patients feel about sensors and AI in healthcare is an important issue. The patients’ perspectives are crucial when considering the use of AI and sensors in healthcare. Interestingly, 35% of patients would refuse the incorporation of intervention using AI and sensors into their care because of its lack of transparency and security. Another study showed that patients were enthusiastic about the ability of AI to be a positive force in healthcare; however, they voiced concern when it came to liability in case of error and a possible increase in healthcare costs.

A 2020 survey of 922 Dutch women aged 16 to 75 years showed that nearly 80% did not support the use of AI for standalone interpretation of screening mammograms, supporting the necessity of a human check. The combination of a radiologist as a first reader and an AI system as a second reader appeared more acceptable. Thus, globally, even if similar concerns are raised, both patients and doctors feel that sensors and AI use should improve care quality [34,35,36].

### 4.2. French Liability Regimens Applying to Healthcare Professionals

Classically, the legal standard of care and medical liability requires investigation of the pathway that lead to the harm suffered by the patient. There seems to be a consensus on the assessment of medical malpractice and compensation for damages [27]. First, there should be an event or a damage to the patient; second, there should be an action that caused the damage; and last, there should be an evaluation of the causal link between the error on the part of the physician and the event (damage) involving the patient [37].

The reconstruction of the actual sequence of events that occurred is part of evaluating a professional liability case, and to do so extensively, it is necessary to analyze the collection of informed consent from the patient, as there cannot be a medical pathway without consent from the patient.

**Figure 5:**
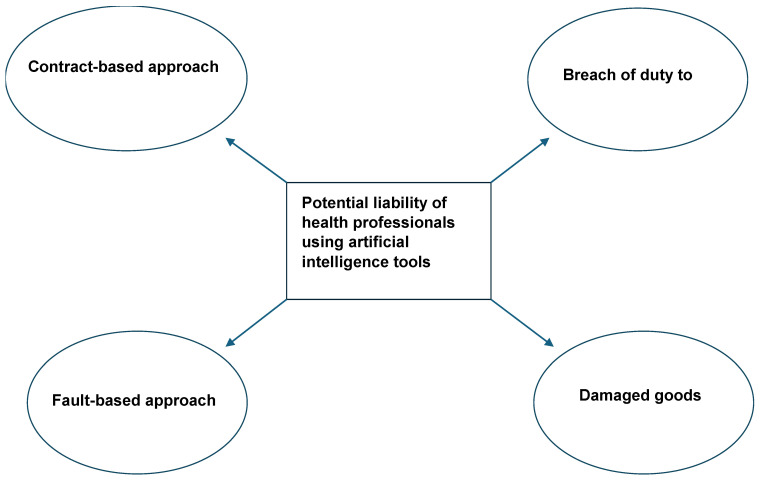
Four situations potentially involving liability of health professionals.

Despite the existing variations in civil and tort law across countries, there seems to be a consensus on the assessment of medical malpractice and compensation for damages. The liability when using sensor and AI tools in healthcare can be analyzed through four approaches: the contract-based approach, the approach based on breach of duty to inform, the fault-based approach, and the approach related to the good itself (Figure 5).

#### 4.2.1. The Contract-Based Approach

A. Context

Contractual medical liability was intended to compensate for damage resulting from the improper performance of a care contract by a healthcare professional. In France, the Mercier decision of the Cour de cassation (Court of Cassation) considered that “a real contract was formed between the doctor and the patient, including several commitments for the practitioner” [38]. In addition to the commitment to use all possible means to cure the patient, it was expected that the physician would provide conscientious and attentive care in accordance with the data acquired from science. The doctor was then considered to be the debtor of an obligation of means. The Act of 4 March 2002 established the principle of liability for fault [39] so that it abolishes the contractual and extra-contractual dichotomy (also known as tort). Thus, there is only legal medical liability, which is based on fault, with the exception of cases of supply of medical devices.

For example, in telemedicine, when damage is caused to a patient as a direct result of malfunctioning telemedicine equipment, the medical professionals involved in the telemedicine act may be held liable in the absence of fault [40]. In this case, they can take recourse action against the technological third party concerned for breach of the obligations set out in the contract between them [41].

B. Proposed Actions Supporting That Care Was Given in Accordance with the Data Acquired from Science

Besides the classical certifications of the doctors and of the sensors generally used, a question may arise as to whether it is pertinent or not to use sensors and AI. Indeed, both sensors and AI might in the future be considered more often as part of the obligation of means, since they have already become part of the standard of care [1,19,20]. Therefore, practitioners should be able to offer up the defense that they did not utilize sensors or AI because the known data from other sources were deemed sufficient to enable adapted care of the patient. In order to further enhance the understanding of the medical professional, better education on sensors and AI systems needs to be developed both in medical and paramedical schools, a process which has already begun. Indeed, even as short as a one-month investment in AI education during medical school would empower physicians, likely supporting better use and innovation in healthcare [42]. Using sensors for remote healthcare is even less time consuming.

Another action made possible when sensors and AI are considered as the standard of care is the setting up of financial incentives to adopt specific autonomous systems. For example, when digital health records became the standard of care, hospitals that did not switch to electronic health records were penalized by insurers [19].

#### 4.2.2. Approach Based on a Breach of the Duty to Inform

A. Context

The French Public Health Code set an obligation for the health profession to provide information, which includes ethical obligations. It lays down the principle that any person must be informed about his or her state of health [43]. This information covers the “various investigations, treatments or preventive actions proposed, their usefulness, their possible urgency, their consequences, the frequent or serious risks normally foreseeable that they entail, as well as the other foreseeable consequences in the event of refusal” [43]. This duty to inform was also stated in the Convention on Human Rights and Biomedicine (ETS No 164) [44], which is the one international legally binding instrument in the biomedical field.

In France, the Act of 4 March 2002 enshrined the changes in case law providing that in the event of a dispute before the court, the burden of proof of compliance with the obligation to provide information lies with the doctor, who may do so by any means. It should be noted that the decisions rendered by the courts and tribunals have strengthened the obligation to provide information. The highest public jurisdiction has recently specified that in the event of omission or insufficiency of information provided by the practitioner, the patient may rely on the failure resulting from this lack of information to seek the liability of the public health establishment before the administrative judge [45]. The same solution was endorsed by the highest civil and criminal jurisdiction of the French judicial system. 

When a decision is made with the help of an automated decision-making system, the professional must inform his or her patient. The professional explains why he or she followed the recommendations and how he or she came to this conclusion with the support of AI.

The caregivers must have the right to refuse to follow AI’s recommendations based on sensors’ parameters of acquisition and analysis if they think it has made a mistake. In the same way, the patient must be free to refuse a decision emanating from sensors coupled to AI systems. In this view, it is noteworthy that AI might help to detect fake news on social media which might be useful to reduce people being mislead as observed recently during the COVID-19 pandemic [46].

B. Proposed Actions Certifying That the Information Was Adequately Given to the Patient

Although not an obligation, it might be useful for the doctor to clearly state and write in the patient’s medical file that sensors and/or AI were used to obtain diagnostic and/or therapeutic options. This is easy to perform, not time consuming, and asked when patients participate in low-risk research protocol. Of course, if a patient takes part in a research protocol evaluating sensors or AI, ethical guidelines must be followed, and informed consent might need to be signed by the patients after ethical approval of the study by an ad hoc committee. It should be highlighted that in order to obtain an informed consent in the context of sensors and AI, patients should be sufficiently informed to “understand risk, benefits and limitations of sensors and AI software” to be able to give consent to their use [47].

Interestingly, rather than an informed consent process change, a modification of patients’ feelings was observed when AI was used in medical care. Despite the increasing use of AI as a clinical decision support, patients might be unaware of AI’s role in the physician’s decision-making process. An interesting recent study centered on patients’ perspectives showed that information regarding the use of AI tools can be perceived as more important than the regularly disclosed information when AI is not involved. Such a perception is modulated by gender, age, and income. These data further reinforce the need to adapt and precisely personify information when AI is used [48].

Informed consent and AI in medicine is a key issue. Importantly, informed consent issues were cited in more than 30% of problematic surgical cases, supporting the need to update the surgical consent process which should accurately reflect the ongoing operation [49]. 

Within the European Union, the General Data Protection Regulation (GDPR) states “patients own and control their own data and must give explicit consent for its use or when it is shared”. In France, the legislation states that the healthcare professional providing care must ensure that the patient has understood the information provided and that he or she is able to give free and informed consent [50]. Thus, the information provided to the patient will not only be about the use of AI but also about how the AI works. In this respect, recent studies demonstrated that when a patient is harmed, a lack of informed consent would result in the weakening of the legal position of the healthcare professional. 

During an investigation into medical harm, the first aspect to be considered is the existence of harm suffered by the patient, which includes gathering detailed patient information, such as medical history, symptoms, tests, and treatments. Then, the healthcare providers’ decision-making processes will be examined regarding diagnosis, treatment, and patient management throughout the medical pathway [51].

Even in cases when no harm results, conducting any medical procedure without obtaining formal consent is unlawful and unethical [52]. 

C. Useful information to share with the patients

Important information includes technical specifications, descriptions of the data algorithms and training processes of AI models in the specific healthcare provided to one patient, and the clinical decision-making process. Such information needs to be simple and adapted to the patient’s general knowledge in order to avoid a black box feeling and to be sure that the consent will really be an informed choice.

Clearly, providing all of the limitations of sensors, telemedicine, and AI without explaining all terms is inadequate. However, the caregiver must adapt to the patient’s ability to understand the information. Thus, the terms used and the technical precision must be comprehensible by the patient. This is already the case today when explaining complicated medical and/or surgical procedures. We will present here some main points that ought to be discussed with the patient.

The technical specifications of sensors and AI such as accuracy, sensitivity, and specificity are crucial to evaluate their effectiveness and should be provided when referring to a specific situation. For example, the evaluation of a novel device using a machine learning algorithm based on movement analysis with four sensors in the setting of diabetic neuropathy demonstrated that the accuracy of the algorithm was 82.1% with a 78% sensitivity and an 87% specificity. This means that the movement conformed to expectations in 82.1% of cases and that potentially 22% of pertinent movements were missed and that 13% were falsely diagnosed as relevant [53]. Sensors with high levels of accuracy in detecting clinically pertinent parameters are now widely used, allowing for remote healthcare in a time of severe shortage of medical and paramedical caregivers [54]. Interestingly, accuracy should not be confounded with efficiency. It is important to distinguish the benefits of AI more clearly in terms of effectiveness (getting more done) and efficiency (doing it with fewer resources) [55]. 

Some selected data on algorithm creation in telemedicine and AI might also be shared with the patients. Regarding the algorithm itself, the concept of AI encompasses mathematic phenomenon from machine learning, deep learning, and natural language processing. Machine learning is the analysis of vast amounts of data, recognition of patterns, the making of predictions based on that data, and even the ability to adjust their behavior accordingly. Meanwhile, deep learning is a subset of machine learning that uses artificial neural networks to learn from data [56]. 

The clinical decision-making process is also important to discuss. Indeed, if well understood, it will lead to a better acceptance of devices’ use. AI and sensors in the medical field are revolutionizing the accuracy and efficiency of healthcare. Notably, a study proposed the use of machine learning algorithms to automatically score Parkinsonian tremors using wristwatch-type wearable sensors, measuring tremor signals from 85 patients with Parkinson’s disease. The results support the feasibility of the proposed system as a clinical decision tool for Parkinsonian tremor-severity automatic scoring [57]. Another study demonstrated that the inaccuracies in heart rate data can be rectified with the sensors, thus ensuring the reliability and precision of the medical devices [58].

Hence, AI sensors in healthcare can help with early disease diagnosis, prompt clinical and caregiver assistance, and ongoing home health monitoring for senior citizens and young adults [49]. 

Finally, information concerning data security might also need discussion. Indeed, when the data collected are not on a secured server, data leakage and potential blackmail can occur both at an individual level and at the level of entire hospitals or even national insurance structures. Greater maturity with health data governance would strengthen the approach and help develop coordinated responses against these threats.

#### 4.2.3. The Fault-Based Approach

A. Context

The French Act of 4 March 2002 provides for liability in the event of fault on the part of the healthcare professional, stating that “the liability of healthcare professionals in respect of acts of prevention, diagnosis or care is incurred only in the event of fault” [59]. In a ruling dated 14 December 2022, the highest civil and criminal jurisdiction (or Cour de cassation) reiterated that, in the event of damage caused during surgery, the healthcare professional is only liable in the event of fault. 

In addition, when there is the question of the medical professional’s fault, in France, the legal arrangement called *perte de chance* has to be taken into consideration. This means that a loss of opportunity occurred, which can happen when it is certain that without the fault, the damage would not have occurred. When there is a loss of opportunity, in the sense that the professional did not mitigate a known risk, then the professional could be held legally liable [60]. For instance, the Court of Cassation criticized a Court of Appeal for having refused to compensate for the consequences of failures attributable to the anesthetist to prevent the risk of arterial hypotension induced by spinal anesthesia [61]. 

As the law currently stands, the doctor remains solely responsible for the treatment of the patient, without prejudice to any recourse or subrogation that the patient may exercise (at a later stage) against the producer of the defective equipment. For instance, in the US in the 2023 case Sampson v. HeartWise Health Systems Corporation [62], physicians followed the output of a software program for cardiac health screening. The Court reversed summary judgment to hear the claim of the wife of the deceased. However, it affirmed in favor of HeartWise considering the lack of evidence proving the fault of the healthcare establishment. Another example was observed in Italy during the COVID-19 pandemic. It related to the current law on the crime of “negligent epidemic” (art. 438, penal code). This law does not provide for exceptions or mitigations for healthcare professionals, potentially implying different scenarios for an act or omission, and the prosecutors at the head of some of the more important judicial districts promoted investigations in this direction, looking for medical misconduct [29].

However, with the emergence of new technologies and of potential sensor- and AI-related medical error, it will be difficult to figure out who is liable. Fault can be attributed to a design defect in the sensor, an “inappropriate use by a healthcare professional, or a lack of maintenance by the hospital” [63]. This is the result of the opacity of new systems “combined with the fact that several players are involved at different stages of the system’s lifecycle”, that renders difficult the identification of those responsible.

In the absence of the recognition of an autonomous legal personality for the algorithm and the robot, it would be conceivable to hold the doctor responsible for the use of artificial intelligence programs, algorithms, and systems, except in the case of a defect in the construction of the machine. For memory, medical malpractice occurs when a healthcare provider causes injury to a patient via negligence or omission in rendering care, and must fulfill four legal criteria: (1) professional duty owed to the patient, (2) breach of that duty via negligent violation of the standard of care, (3) negligence resulted in injury, and (4) injury resulted in damages. 

A special mention can be discussed in the setting of robotic surgery, since such techniques are increasingly used worldwide and are thought to be associated with increased risks. 

In this view, very interestingly, the Westlaw legal database including 25 US states, highlighted that malpractice claims involving robot-assisted surgical procedures increased more than 250% in the past 7 years compared to the previous seven years. Among 45 cases, defendant verdicts predominated (77.8%), with only four plaintiff verdicts (8.9%) and six settlements (13.3%). However, the most frequent liabilities claimed were not directly related to robotic procedures. Negligent surgery (82.2%), misdiagnosis/failure to diagnose (46.7%), delayed treatment (35.6%), and lack of informed consent (31.1%) were the most common abnormalities.

Whatever the surgical field analyzed, litigation in robot-assisted surgery is not frequent, and claims are mainly related to the medical management of the patient. Better informed consent, credentialing, and continuing medical education will help to improve surgeons’ confidence, minimize litigation, and ultimately provide safer and better care for patients [49,64]. Accordingly, a decline in liability claims now occurs, likely resulting from increased training for early adopters of robotic surgery, a cohort known to be at highest risk of litigation among surgeons performing robot-assisted urologic procedures [29].

On the other hand, eventually, doctors or surgeons not using technology (robotic or not) that is demonstrating great results could be sued for negligence. 

Indeed, AI devices offer enhanced diagnostic accuracy by identifying subtle patterns and recognizing specific symptoms that may be overlooked by human practitioners [65]; thus, they are becoming more and more a part of the standard of care for the medical professional working with them. So, for a doctor not to be using technology that has shown great results could in the future be seen as negligence. A study showed that surgical robots were shown to result in better-than-conventional surgical procedures in prostatectomy. It was demonstrated that “robotic-assisted radical prostatectomy offered fewer biochemical recurrence and improvement in quality of recovery and pain scores only up to 6 weeks postoperatively compared to open radical prostatectomy” [66]. This resulted in the incorporation of the AI device into the medical practice of the clinic.

B. Actions to Precisely Define the Responsibility of the Involved Caregiver

As far as possible, when sensors and/or AI are used, there should be a document precisely defining the responsibility of each participant. For example, remote telemonitoring has been widely used during the COVID-19 pandemic in order to obtain early hospital discharge, allowing beds to be saved for other patients, and it will again be used to follow the numerous patients suffering from long COVID [11,67,68,69]. This is a simple use case, but doctors would have been responsible if one parameter (blood saturation decrease related to worsening of pneumopathy secondary to virus spreading) were not to be detected or not transmitted early enough to the doctor. In this situation, it might be useful to state that when the doctor does not respond to an alarm, it should be considered that the professional was not aware of the patient’s potential clinical degradation. The other caregiver acting amongst of the medical chain (whether an engineer, a nurse, or another health professional) should hold the responsibility for such information. In the field of radiology, liability of the professional will be related to the degree of autonomy of the sensor/AI device. For example, when it is used only as a decision support, the radiologist who makes the final determination would be the one bearing the liability risk. However, when the sensor/AI algorithm acts autonomously, it could be “considered analogous to an employee of a facility, its negligence could be attributed to its supervising radiologist or to the institution” [47]. Of course, similarly to the example provided just before in telemedicine, a radiologist would be held liable if he/she had the chance to review the report and to detect errors and thus, the patient’s injury might have been prevented. 

In order to precisely identify the one responsible when liability issues arise, it is crucial that the medical professional no longer views sensors and AI system as a “black box”. Indeed, the black box [70] signifies that sensors and AI are not understood by human intelligence [65] and thus will not be deemed trustworthy by the professional. To combat this lack of knowledge on sensors and AI algorithms, there is an important need to explain their abilities, beginning from the starting parameters and going through to the decision taken by the tool. However, this does not mean that medical professionals should become mathematicians or experts in algorithms, because that would be senseless. The idea is to get to know and understand sensors and AI better, and to get doctors to think along with tech people, to build a productive dialogue.

Finally, a legal solution to liability could be the conferral of personhood to sensor-coupled AI devices. This would result in direct lawsuits against them in cases of malpractice. Similarly to what currently exists with some tech devices, the sensors/AI users would be able or forced to have a liability insurance that would make sure that they can provide compensation if harm arises. Thus, it could help resolve the issue of the lack of accountability in sensors use in healthcare.

#### 4.2.4. The Approach Related to the Good Itself

A. Context

There are two other liability regimens derived from fault-based liability which should be brought up when discussing medical liability: the liability for the action of the thing and the liability for damaged goods.

Liability for damage caused by things involves the liability of a person who causes damage to another person with a thing “of which he had the use, direction and control at the time of the damage” [11]. Liability for damage caused by things presupposes an act on the part of the thing causing the damage and finds its legal basis in Article 1242 of the French Civil Code. This system of liability has been widely used in jurisprudence relating to liability for robots, since robots and AI act in the health and medico-social field solely under the care of a human being. Now, with the emergence of autonomous sensor-based AIs, the relationship seems to be reversing, as the professional can be tempted to follow the recommendations of the system, even for the diagnosis of patients.

Regarding such liability, an example from American case law is interesting. The plaintiff sued a clinic and its doctor who relied on the output of a software program for heart evaluations to determine whether they had inherited a heart defect. Here, the Court judged against the plaintiff, as they failed to present substantial evidence that the course of conduct of the deceased would have changed if he had not been at the clinic [71]. Nonetheless, importantly, with substantial evidence, the doctor could have been held liable after following a incorrect output of an AI device.

Liability for defective products is the obligation incumbent on the producer, manufacturer, distributor, seller, or lessor of a good to repair the damage caused by a good that does not offer the safety that can legitimately be expected. It applies to products put into circulation after 21 May 1998, and to damage exceeding 500 euros but concerns only personal injury and death. Thus, this liability regime could apply to sensors and AI systems released as a product (tangible tool) and not as a service (software). However, the producer may escape liability if he or she demonstrates that, at the time the product was put on the market, the state of scientific knowledge did not allow one to detect the defect [72]. This exemption is neutralized when “the product is a component of the human body or a product derived from it” [73]. However, it is extremely difficult to define what constitutes an actual defect because of some unpredictability involved in sensor-based AI systems. Interestingly, concerning robotic surgery, device failure was rarely cited. 

It has to be noted that for the first time, the highest civil and criminal jurisdiction retained a joint liability of the prescriber and of the producer. In this case from 2023, the product was defective because the information on the patient leaflet was inadequate, and the medical professional ended up being held liable for failing to properly inform his patient of the risks of the treatment, since, as a professional, he had access to information that the patient could not have had.

Thus, these two regimes are interesting by their very nature, but they would only apply restrictively to certain tools. In this digital era, it must be understood that a sensor/AI couple is not “one technology but a heterogeneous group with varying liability risks” [74]. In this regard, systems are no longer things in the sense of tangible products but can be services. As a result, French law is currently unable to respond to the problems faced by doctors who have delegated an act to sensors and/or AI which their hospital has imposed on them and of which they know neither the precise functioning nor the relevance.

Whether liability changes when an AI system makes a mistake can be questioned. In practice, there should be different liability rules for different risks. When the AI is in a non-autonomous device such as a surgical robot, the liability of the surgeon could be involved. In France in 2019, the Tribunal of GRASSE held that, in the case of damage suffered by the victim of an error on the part of the health professional during an operation, “when it is attributable to several persons acting independently, the victim may seek compensation for her/his damage” [75]. In this configuration, a health professional using an AI device would be held liable conjointly with the manufacturer if an error occurred.

Regarding the specific deployment of wearable devices/sensors, a study proposed a specific management model involving initial human intervention by a nurse. In the event of an alarm by a sensor, the nurse might decide whether to continue monitoring (e.g., in the event of a false alarm) or to alert the ward physician when the AI device is not making a mistake. This would result in a form of shared liability where each professional could be identified and held accountable for her/his action. 

B. Actions Aiming to Reduce Liabilities for the Action of Things or for Damaged Goods

First, the health authorities must validate all sensor devices before use. Then, the practitioner or the hospital should continuously follow declarations of undesirable events. This is true both for materials, such as robots, but also for software—not only software characterizing the AI’s functioning but also software keeping the medical secret inviolable. This is one of the main concerns making doctors reluctant to use sensors and AI.

Regarding the medical devices, including software, they have to comply with the local medical device regulations. For example, in the EU, regulations call for CE marking and, in the USA, for clearance or approval by the FDA [20]. Additional frameworks exist in other countries, such as in Japan through the Pharmaceuticals and Medical Devices Agency or in the UK, where the Medicines and Healthcare Products Regulatory Agency has taken over responsibilities previously handled by the EU. It should be stressed that in 2022, the FDA cleared only 92 medical devices, when 600 articles are presented each year [65].

Finally, having different responsibilities at different stages of the sensor/AI system’s lifecycle seems to be a functioning approach used in the EU [32], particularly when there is not one piece of technology but a heterogeneous group with varying liability risks. It was mentioned in the article that there would be new regulation created specifically in the context of sensor/AI systems in healthcare, which could be a good way to provide a better framework, especially when it has been proven that liability laws can “encourage adoption of technologies that reduce harm (to users, workers, or the public)” [60].

## 5. Legal Aspects and Liability in Different Countries

To complete the data previously reported, we emphasize here that there are different specific laws related to AI and healthcare depending on the country. Thus, the challenges and responsibilities of healthcare professionals vary when using AI technology. Ethical views might also depend on the social and cultural background of the authority establishing the rule. In Europe, the EC and the Council of Europe have established guidelines regarding ethical AI in healthcare. An important issue to be highlighted is the explainability that improves the trustworthiness of the AI applications and allows for the uncovering of potential biases in the AI models [65]. Further, patients’ right to privacy and the security of the data are paramount ethical responsibilities. 

The European regulation (AI Act) distinguishes the roles and responsibilities of AI developers (providers) and users (deployers) [14], while the European regulation MDR sets the manufacturer as the entity responsible for the device and imposes extra obligations regardinginstructing users and avoiding user errors [76].

In California, the tort plaintiff of a car accident established that a coding error caused the operating system to crash. This was then considered to be a ‘malfunction’ that subjected the manufacturer to strict tort liability [77]. Such reasoning could apply regarding the use of AI systems and sensors in healthcare if there is a malfunction of the device itself.

In South Africa, the common law imposes fault-based liability on the human healthcare practitioner, which entails that “one may be held liable if one fails to meet the objectively measured standard expected of a reasonable practitioner in his/her branch of the profession” [78]. However, this fault-based approach seems unaligned with the African tradition (the idea of reconciliation instead of litigation [79]) and with the WHO guidelines considering that liability is not only about establishing fault. More often, it is about “owing to the nature of the technology and making sure there is human accountability—either through sole, or joint and several, liability”.

## 6. Conclusions and Perspectives

We investigated different aspects of the liability of health professionals using sensors and AI in healthcare. Besides reporting the legal context and practical applications of four common regimes (the contract-based approach, the approach based on breach of duty to inform, the fault-based approach, and the approach related to the good itself), we added knowledge from different real-life cases from different countries.

Good technical characteristics such as accuracy, sensitivity, and specificity on one hand and adequation of the sensors and AI algorithms to a specific disease on the other hand are needed to avoid mistakes. Indeed, for example, even if it was felt that COVID-19 catalyzed willingness to adopt AI by ophthalmologists, some AI devices developed for diagnosing, triaging, and predicting the progression of disease demonstrated inadequate accuracy and predictive value [80,81]. Insufficient quality of data highlighted the need for further validation of AI technologies in healthcare, especially in public health crises like the COVID-19 pandemic.

Further, regarding the interdisciplinary approach, it is important to consider that AI devices regroup various technologies falling under the scope of both international and/or national legislation. Overall, the policies surrounding responsibility for errors committed by AI systems are still developing, and the determination of liability relates to the type of error, the responsibilities amongst stakeholders, and the existing legal frameworks.

The degree to which a health professional may be legally responsible for an AI algorithm’s error depends on how the tool is integrated into the medical standard of care and on the algorithm’s autonomy.

In brief, multiple regimes coexist to apprehend the liability of healthcare professionals. With new types of sensors and AI that are increasingly autonomous, difficulties arise when looking to the attribution of liability. In the current framework, there is a sort of shared responsibility that falls with the degree of implication of the professional, whether it is a fault based on the action or negligence.

In the future, there will certainly be new rules surrounding sensor devices and AI liability, as it is currently debated whether legal personhood should be granted to “autonomous” entities.

Therefore, healthcare organizations should anticipate the problems that may arise by preventing injuries with the creation of a true sensor/AI culture within healthcare professionals and by working to create ethical systems. Some existing frameworks regarding AI use (e.g., autonomous cars) would be useful for drawing parallels to healthcare.

Indeed, the lack of adequate legal protection for healthcare professionals and patients may significantly decrease the care provided to patients.

## Data Availability

All articles cited are freely available.
